# Evidence of horizontal urban heat advection in London using six years of data from a citizen weather station network

**DOI:** 10.1088/1748-9326/ac5c0f

**Published:** 2022-03-24

**Authors:** O Brousse, C Simpson, N Walker, D Fenner, F Meier, J Taylor, C Heaviside

**Affiliations:** 1 UCL Institute for Environmental Design and Engineering, The Bartlett Faculty of Environment, University College London, London, United Kingdom; 2 Bioengineering Sciences Research Group, Department of Mechanical Engineering, School of Engineering, Faculty of Engineering and Physical Sciences, University of Southampton, Southampton, United Kingdom; 3 Chair of Environmental Meteorology, Institute of Earth and Environmental Sciences, Faculty of Environment and Natural Resources, University of Freiburg, Germany; 4 Chair of Climatology, Institute of Ecology, Technische Universität Berlin, Germany; 5 Department of Civil Engineering, Tampere University, Tampere, Finland

**Keywords:** citizen weather station, LCZ, London, urban heat advection, urban climate, crowd-sourcing, Netatmo

## Abstract

Recent advances in citizen weather station (CWS) networks, with data accessible via crowd-sourcing, provide relevant climatic information to urban scientists and decision makers. In particular, CWS can provide long-term measurements of urban heat and valuable information on spatio-temporal heterogeneity related to horizontal heat advection. In this study, we make the first compilation of a quasi-climatologic dataset covering six years (2015–2020) of hourly near-surface air temperature measurements obtained via 1560 suitable CWS in a domain covering south-east England and Greater London. We investigated the spatio-temporal distribution of urban heat and the influences of local environments on climate, captured by CWS through the scope of Local Climate Zones (LCZ)—a land-use land-cover classification specifically designed for urban climate studies. We further calculate, for the first time, the amount of advected heat captured by CWS located in Greater London and the wider south east England region. We find that London is on average warmer by about 1.0 ^∘^C–1.5 ^∘^C than the rest of south-east England. Characteristics of the southern coastal climate are also captured in the analysis. We find that on average, urban heat advection (UHA) contributes to 0.22 ± 0.96 ^∘^C of the total urban heat in Greater London. Certain areas, mostly in the centre of London are deprived of urban heat through advection since heat is transferred more to downwind suburban areas. UHA can positively contribute to urban heat by up to 1.57 ^∘^C, on average and negatively by down to −1.21 ^∘^C. Our results also show an important degree of inter- and intra-LCZ variability in UHA, calling for more research in the future. Nevertheless, we already find that UHA can impact green areas and reduce their cooling benefit. Such outcomes show the added value of CWS when considering future urban design.

## 1. Introduction

Recent studies have highlighted growing interest and opportunities for urban climate studies using crowd-sourced urban meteorological data (Steeneveld *et al* 2011, Wolters and Brandsma 2012, Muller *et al* 2015, de Vos *et al* 2020). Among a variety of crowd-sourcing devices, citizen weather stations (CWS)—also sometimes referred to as personal weather stations—have been gaining popularity. Since their evaluation against official automatic weather stations measurements (Bell *et al* 2015), CWS were sought to help measure urban temperatures in large cities (Chapman *et al* 2017, Meier *et al* 2017). In fact, CWS increase the potential for improved geographical coverage of observations in cities, rather than only relying on established official meteorological stations, which are often lacking in numbers within cities and therefore in representation of urban climate features (Oke 2004, Grimmond 2006, Muller *et al* 2013). Densifying CWS networks not only improves our understanding of the urban climate but could further help, for example, intelligent designs of greenspace, street shading and ventilation, surface materials and buildings, and heat adaptation tools (Goodess *et al* 2021). For instance, the monitoring of locations at higher chances of heat stress in real-time using CWS data (Varentsov *et al* 2020) illustrates the range of applications offered by CWS. Although CWS are subject to greater uncertainties, quality-checking procedures for air-temperature data exist either based on CWS biases against official automatic weather stations located at a certain distance (Meier *et al* 2017, Hammerberg *et al* 2018) or on statistics among CWS alone (Napoly *et al* 2018, Fenner *et al* 2021).

The potential applications of CWS are wide and continue to expand. For example, recent studies (e.g. Fenner *et al* 2017, Benjamin *et al* 2021, Potgieter *et al* 2021, Varentsov *et al* 2021) have used CWS to improve our understanding of the impact of land-use and land-cover on urban temperatures through the perspective of Local Climate Zones (LCZ; Stewart and Oke 2012)—a land-use and land-cover classification specifically developed for urban climate studies. Others have used CWS to validate urban climate simulations (Hammerberg *et al* 2018), drive indoor-temperatures in urban climate simulations (Jin *et al* 2021), and model air temperatures in European cities using machine learning (Venter *et al* 2020, 2021, Vulova *et al* 2020, Zumwald *et al* 2021). Research using CWS has not been limited to urban temperature; some have used them to monitor (urban) precipitation (de Vos *et al* 2019, 2020) or wind speed (Droste *et al* 2020)—allowing for the development of an innovative quality-check for crowd-sourced wind data (Chen *et al* 2021).

Most research at the time of writing has focused on short time periods that do not extend beyond a year and usually focus on summer periods only. This means that analyses of multiple years climatology have not yet been performed. Nonetheless, Meier *et al* (2017) and Fenner *et al* (2017) both studied the whole year climatology in the city of Berlin using CWS and characterized some intra-urban variability of urban temperatures among different LCZ. Additionally, Fenner *et al* (2019) used CWS to study the variability of urban heat island intensities during heatwave events in the extended summer season (May–September) of 2015–2018. They did not, however, look at the winter. Apart from Venter *et al* (2021), who studied urban heat islands among multiple European cities for one summer month in 2019, large-scale analyses (e.g. regional, national or continental) have also not been extensively performed. As such, most of the studies focus on single cities only. Lastly, while some studies have already investigated the weather-dependent variability of the urban heat islands using CWS (e.g. Chapman *et al* 2017), none have yet studied how certain prevailing winds may cause horizontal urban heat advection (UHA) and the sensitivity of CWS measurements to this advected heat.

In fact, previous studies show that UHA impacts the spatial distribution of urban heat (Heaviside *et al* 2015, Bassett *et al* 2016). UHA can be considered as the heat resulting from the windborne transport of energy amongst the urban environment (Oke *et al* 2017). It is often neglected because modelling and observational studies of urban heat tend to assume that horizontal diffusion is similar across the urban landscape and that major heat production comes from differences in surface energy balance related to land-use/land-covers. Nevertheless, a modelling study of the August 2003 heatwave in the city of Birmingham, England, Heaviside *et al* (2015) showed that UHA can reach up to 2.5 ^∘^C, particularly under north-westerly and south-easterly wind conditions. Using a dense network of weather stations in the same city, Bassett *et al* (2016) showed a mean UHA over a 20-months period (January 2013–September 2014) of 1.2 ^∘^C, and argued that it could be higher under certain specific conditions as shown in Heaviside *et al* (2015). This demonstrates that UHA can have a significant impact on urban temperatures, and needs to be properly understood to develop future urban heat adaptation and mitigation strategies in cities that include the potential harmful effects of temporally varying UHA in their designs.

In London, England, CWS density has continuously been growing over recent years and urban heat island intensity has not changed over recent decades (1950–2019; Bassett *et al* 2021). Besides, the urbanization rate has been steady and rather slow in recent years, being around 1% per decades (Bassett *et al* 2020). This makes it a suitable place to study urban heat heterogeneities and horizontal advection over recent years. This study thus makes a first attempt to demonstrate how CWS can aid urban heat advection studies, by capturing complex dynamical and physical processes in urban environments. Therefore, in this study we: (a) build a 6-year quasi-climatology—referring to a climatology shorter than 30 years covering multiple full years with all seasons—of near-surface air temperature, from 2015 to 2020, by acquiring, quality-checking and filtering all CWS present in a large domain covering southeast England and the Greater London area; (b) use this quasi-climatology to look at the seasonal heterogeneity of urban heat in the domain and relate it to the locations of the CWS and the underlying LCZ land-use/land-cover; in order to (c) define an appropriate domain to study and quantify the amount of UHA measured by CWS in each LCZ over the Greater London area in different seasons, and depending on prevailing wind speed and direction. This study is the first to investigate UHA and related urban heat heterogeneities among different LCZ using CWS in Greater London. Hence, to continue this effort, we provide recommendations for future research at the end of the manuscript.

## 2. Methodology

### 2.1. Study area and period

**Figure 1.**
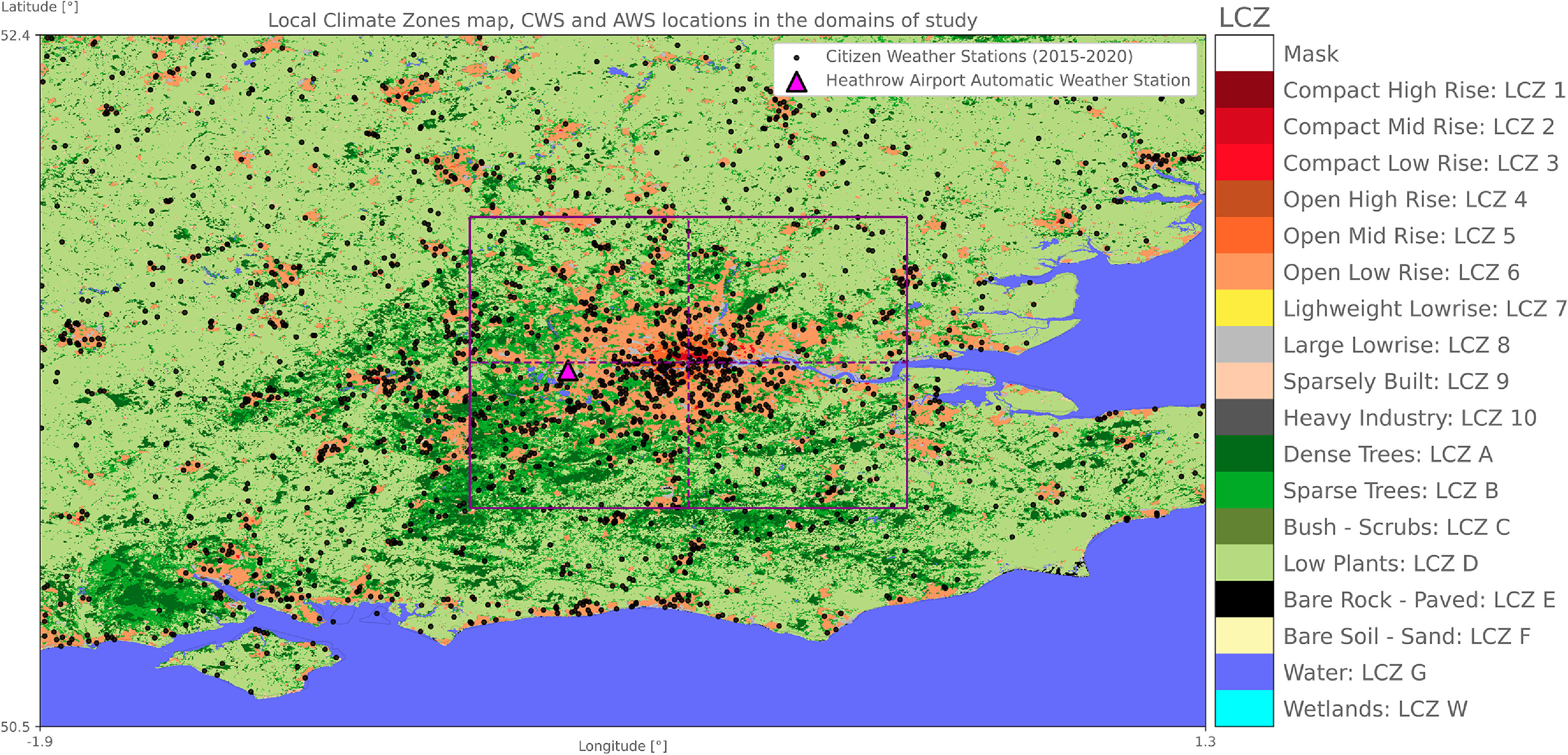
Map of Local Climate Zones (LCZ) in the larger domain of study (domain 1) at 100 m horizontal resolution. The smaller domain (domain 2) is shown in purple with dashed lines representing the separation between four geographical quadrants for the UHA analysis. Black dots represent the location of quality-checked Netatmo Citizen Weather Stations (CWS) available within the time period from year 2015 to 2020. The Heathrow Airport MetOffice automatic weather station location is shown in fuchsia. LCZ source: Demuzere *et al* (2019).

Our study is focused on a large domain covering southeast England (figure 1), which includes the Greater London Authority administrative area and its surrounding urban areas. This large domain is host to 24 million inhabitants out of which 4 million are aged above 65 years old, and therefore are potentially more vulnerable to the affects of heat (Office for National Statistics 2021). In the smaller domain, there are 13 million inhabitants and 1.7 million are older than 65 years old. This makes it a relevant case-study for urban temperature monitoring. We define our 6-year study period from 2015 to 2020.

We use two domains to perform two types of analyses at varying spatial and temporal scales (figure 1). First, an extended domain (Domain 1) covering the Greater London area and the secondary urban nuclei in all directions—extending from 1.9^∘^ W to 1.3^∘^ E and 50.5^∘^ N to 52.4^∘^ N—is used for the data collection and for studying the regional climate to define a second domain used to study the UHA. This analysis is provided in supplementary section S4 (available online at stacks.iop.org/ERL/17/044041/mmedia). Domain 2—subjectively derived after analyzing the spatial distribution of averaged temperatures in Domain 1 (see supplementary section S2)—is centered on 0.12^∘^ W and 51.5^∘^ N, close to Trafalgar Square, and extends by 0.6^∘^ in west and east directions and by 0.5^∘^ in north and south directions (figure 1). An in-depth study on the influence and seasonality of prevailing winds on UHA is performed within Domain 2. To study hourly UHA, the domain is divided in four quadrants—North-East (NE), North-West (NW), South-West (SW), and South-East (NE) (see section 2.3 for the definition)—following Heaviside *et al* (2015) and Bassett *et al* (2016). The study uses a clipped part of the European Local Climate Zones (LCZ) map by Demuzere *et al* (2019) (see supplementary information S1 for more information on LCZ).

### 2.2. Data collection, filtering and normalization

**Figure 2.**
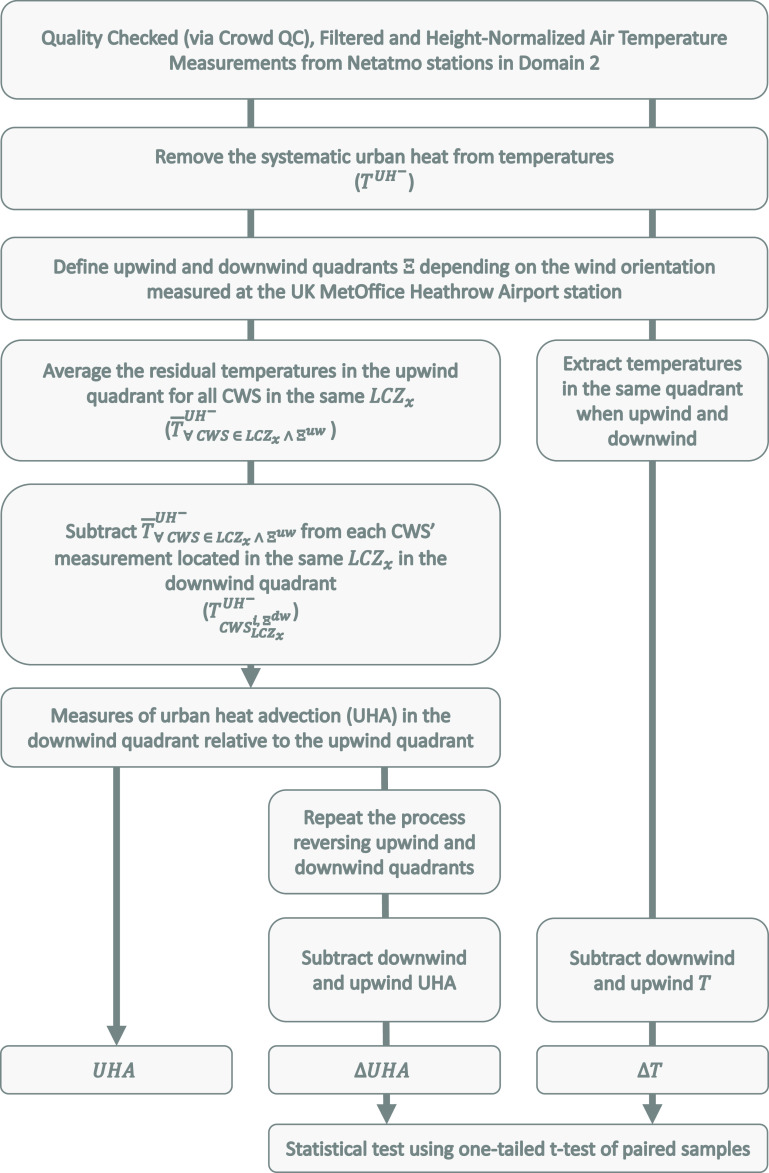
Flowchart diagram showing the different steps undertaken to calculate the downwind urban heat advection (UHA), the upwind-downwind gain/loss heat anomaly ΔUHA and the upwind-downwind temperature anomaly ΔT.

In this study, two types of weather stations are used: Netatmo CWS, used for acquiring crowd-sourced measurement of hourly near-surface air temperature, and the Heathrow official MIDAS monitoring station from the United Kingdom Met Office (UKMO 2021), used for hourly observations of wind speed and direction. The whole methodology is summed up in a flowchart diagram that is given below (figure 2).

#### 2.2.1. Air temperature measurements: Netatmo citizen weather stations

Netatmo CWS consist of two cylindrical modules shaded and protected by a cylindrical aluminium shell. Both modules, consisting of an outdoor and an indoor module, measure air temperature and relative humidity. The indoor module additionally measures CO_2_ concentrations, air pressure and noise levels (Meier *et al* 2017). We collected hourly data from all Netatmo stations within Domain 1 for the whole 6-year period by using the *Getpublicdata* and *Getmeasure* functions of the Netatmo company’s (https://netatmo.com) API (https://dev.netatmo.com/). We quality-checked the measurements using CrowdQC v1.2.0 R package developed by Grassmann *et al* (2018) and used in Napoly *et al* (2018). We consider only CWS measurements passing the M4 quality-check level. The M4 quality-check level filters out hourly measurements: (a) taken by duplicated stations (same coordinates); (b) that are considered outliers based on their z-score compared to the other measurements; (c) of a whole month if more than 20% of the measurements in that month were removed in the previous steps; and (d) if the measurements are considered indoors by not being sufficiently correlated to the median temperature of all measurements (Pearson *r* $\lt$ 0.9). This results in a reduction of the CWS sample from 1783 potential stations to 1560 suitable stations. Then, we keep only CWS measurements when at least 80% of a year witthe whole 6-year period is available, following Fenner *et al* (2019). This reduces suitable CWS down to 884 in Domain 1. 423 are located in Domain 2. We do not consider the remaining single station in compact low-rise (LCZ 3) as it cannot be used for UHA quantification. More information on the quality-checking and the distribution of CWS among the domains and LCZ is given in supplementary section S3, figures S2–S4 and tables S1–S3.

In this study, we consider each CWS to be representative of the LCZ it is in. We chose to perform this simplification to avoid losing additional data after performing our quality-check and filtering. Nevertheless, micro- and mesoscale effects are known to affect measurements in air temperature at local scales (Fenner *et al* 2017, Skarbit *et al* 2017, Quanz *et al* 2018, Varentsov *et al* 2021)—something that has to be kept in mind in our further analysis. Besides, we normalize the temperature observations by height, following Potgieter *et al* (2021), to get rid of the vertical thermal gradient. 0.0065 ^∘^C are hence summed to the observed temperatures per meter anomaly to the average height across the domain. The average elevation of all CWS, obtained from the Shuttle Radar Topography Mission (SRTM) elevation product at 30 m horizontal resolution, is 62.3 m.

#### 2.2.2. Wind speed and direction: MIDAS automatic weather stations

We gathered measurements of wind speed and direction from the Heathrow Airport official Met Office automatic weather station (UKMO 2021) to define hourly prevailing winds over the Greater London area for the same 6-year period as the Netatmo data (2015–2020). This station follows the World Meteorological Organization standards and offers measurements of average wind speed and direction at hourly time steps at 10 m above ground level (Sunter 2021). It is a commonly used official station for climate studies focusing on London and surroundings (Mayes 2021). Besides, the Heathrow station is located at a similar latitude to the center of the Greater London area and is sufficiently close to the urban area to be considered representative of winds affecting the Greater London area. It is also one of the only stations in the Greater London area that cover the whole period of interest. We convert its wind speeds measurements from knots to meters per second by considering: 1 kt = 0.5144 m·s^−1^.

It is important to note that few other stations in the Greater London area also capture wind data for our period of interest: Kew Gardens, Northolt or Kenley Airfield. Our choice to only use the Heathrow station for defining prevailing winds is an important caveat of this study, since wind orientation can vary importantly across the urban environment. Nonetheless, testing the representativity of official weather measurements to characterize prevailing winds in cities is not the purpose of this study. It should therefore be considered as a first analysis on the potential of CWS to quantify UHA using a standardized method that must be ameliorated in the future (see the discussion section below).

### 2.3. Wind regimes definition

To study the impact of wind speed and direction on the seasonal intra-urban heterogeneity of air temperature we classify wind speed into four easily understood categories with bins of 3 m·s^−1^, namely: *Calm or Light Breeze* with positive wind speed below 3 m·s^−1^; *Gentle to Moderate Breeze* with wind speed from 3 m·s^−1^ to 6 m·s^−1^; *Moderate to Fresh Breeze*, from 6 m·s^−1^ to 9 m·s^−1^; and *Strong Breeze* with wind speed above 9 m·s^−1^. We chose the upper category of 9 m·s^−1^ and above since less than 0.5% of the winds within our 6-year period are at speeds higher than 12 m·s^−1^ (figure S3) and because it is close to the 95$\textrm{th}$ percentile. Over the six years, the median speed is 3.6 m·s^−1^, the mean speed is 4.2 m·s^−1^ and the maximum observed hourly wind speed is 18.5 m·s^−1^. 31.6% of the available hourly wind measurements are considered as *Calm or Light Breeze*, 48.59% as *Gentle to Moderate Breeze*, 16.19% as *Moderate to Fresh Breeze* and 3.63% as *Strong Breeze*. In general, the winds follow a log-normal distribution and our simplified classes therefore cover meaningful probabilistic distributions with the first two covering common events, whilst the second two focus on more extreme situations with different occurrence probabilities.

### 2.4. Measuring urban temperatures and urban heat advection

Since the majority of the CWS are located within urban built environments (table S2), and since we cannot ascertain that CWS located in natural LCZ are not influenced by urban heat, we decided to focus on air temperature and daily temperature ranges instead of quantifying the urban heat island intensity. This idea follows the recommendations provided by Stewart (2011) and Stewart and Oke (2012), who argue that urban climate studies should focus more on the quantification of urban heat than on the urban heat island intensity (also see Martilli *et al* 2020).

To study the effect of wind speed and direction on the inter- and intra-LCZ heat heterogeneity, hourly values are used to avoid compensating effects that may occur, for example if the wind direction changes significantly within a 24 h period. We define UHA as the air temperature anomaly measured by the CWS between an upwind and a downwind quadrant that is not related to the land-use land-cover and environmental differences between the two quadrants (equation (1)). Our Domain 2 quadrants, hereafter named Ξ, are defined by angles of 90^∘^ increasing clockwise. Importantly, the upwind and downwind quadrants, $\Xi^{uw}$ and $\Xi^{dw}$, respectively, change when wind direction changes. Hence, for example, the North-Eastern and the South-Western quadrants are considered upwind and downwind, respectively, when winds are blowing from angles between 0^∘^ and 90^∘^. The opposite occurs if winds are blowing from angles between 180^∘^ and 270^∘^. We do not include hours when wind direction recently changed by filtering out hours when the wind direction is not blowing from the same quadrant for at least three hours—the first two hours are hence excluded. This threshold is subjectively fixed based on the distance from the center of each quadrant to the center of Domain 2 and the median wind speed over the six years. We also filter out hours with wind speed equal to 0 to avoid accounting for hours when no urban heat is advected. This reduced the amount of potential studied hours by 30% without importantly affecting the probabilistic distribution of wind regimes (see supplementary section S3).

Once this filtering is done, we test the statistical significance of measured UHA by CWS in two steps: first, we test how averaged winds generally affect each quadrant by testing with a one-tailed t-test of paired samples whether the averaged air temperature in these quadrants is significantly lower when located upwind than downwind—we call this difference ΔT; second, we test how prevailing winds are responsible for heat advection in the urban area by testing with a one-tailed t-test of paired samples whether the same quadrant when located upwind or downwind significantly loses or gains heat, respectively, in comparison to the opposite quadrant—we call this ΔUHA (equation (2)).

To measure UHA (and hence ΔUHA) we consider the strategies adopted by Heaviside *et al* (2015) and Bassett *et al* (2016) to measure the hourly advected heat under different wind regimes. Our method, however, diverges from theirs as Heaviside *et al* (2015) used a non-urban and an urban climate simulation to quantify the expected average urban heat in each quadrant, and Bassett *et al* (2016) normalized the observed average urban heat of each quadrant by their urban fraction. Here, we assume that similar LCZ are expected to have a similar impact on the local urban heat anomaly. In fact, LCZ are by definition ‘regions of uniform surface cover, structure, material, and human activity that span hundreds of meters to several kilometers in horizontal scale’ (Stewart and Oke 2012).

To quantify the two-dimensional advected heat at each CWS location per LCZ ($\overline{UHA}_{\mathrm{CWS}_{\mathrm{LCZ}^{x}}^{i, {\Xi}^{dw}}}$), we first have to ascertain that the differences in urban heat are not related to the local land surface characteristics responsible for the averaged UHI, nor to the average UHA related to the location of the CWS at which advection is measured. The time-mean average temperature per CWS ($\overline{T}_\mathrm{CWS}$) is subtracted from the hourly measured temperatures prior to calculating the UHA, resulting in temperatures independent of systematic urban heat ($T^{UH^{-}}$). We then subtract the average temperature of all CWS located in a certain LCZ from the upwind quadrant ($\overline{T}_{{\forall}~\mathrm{CWS}~{\in}~{\mathrm{LCZ}^{x} \land {\Xi}^{uw}}}^{UH^{-}}$) to each CWS of the same LCZ located in the downwind quadrant ($T_{\mathrm{CWS}_{\mathrm{LCZ}^{x}}^{i, {\Xi}^{dw}}}^{UH^{-}}$; equation (1)). This way we look at the additional UHA related to each prevailing wind event rather than to the average UHA and UHI at the location. As Heaviside *et al* (2015) note, ‘this assumption is reasonable if statistical distributions of meteorological quantities are independent of wind direction’. According to the method given above, we hence have three metrics of importance in the analysis: UHA, ΔUHA and ΔT. They are calculated as such:

\begin{equation*} \overline{UHA}_{\mathrm{CWS}_{\mathrm{LCZ}^{x}}^{i, {\Xi}^{dw}}} = \overline{T_{\mathrm{CWS}_{\mathrm{LCZ}^{x}}^{i, {\Xi}^{dw}}}^{UH^{-}} - \overline{T}_{{\forall}~\mathrm{CWS}~{\in}~{\mathrm{LCZ}^{x} \land {\Xi}^{uw}}}^{UH^{-}}} \end{equation*}

where $\overline{UHA}$ is the average advected heat at $i\textrm{th}$ CWS, located in *x* LCZ in the downwind quadrant $\Xi^{dw}$ during certain prevailing wind conditions. $T^{UH^{-}}$ is the hourly temperature of each CWS located in *x* LCZ of the smaller domain minus the time-mean average temperature of the same CWS ($T_\mathrm{CWS}^{UH^{-}} = T_\mathrm{CWS} - \overline{T}_\mathrm{CWS}$), while $\overline{T}_{{\forall}~\mathrm{CWS}~{\in}~{\mathrm{LCZ}^{x} \land {\Xi}^{uw}}}^{UH^{-}}$ is the average temperature deprived of background urban heat ($UH^{-}$) of all CWS located in *x* LCZ and in the upwind quadrant $\Xi^{uw}$. All average temperatures $\overline{T^{UH^{-}}}$ are normalized by the height of the CWS.

\begin{equation*} {\Delta}\overline{UHA}_{\mathrm{CWS}_{\mathrm{LCZ}^{x}}^{i, {\Xi}^{q}}} = \overline{UHA_{\mathrm{CWS}_{\mathrm{LCZ}^{x}}^{i, {\Xi}^{q}}}^{{\tau}^{uw}} - UHA_{\mathrm{CWS}_{\mathrm{LCZ}^{x}}^{i, {\Xi}^{q}}}^{{\tau}^{dw}}} \end{equation*}

where ${\Delta}\overline{UHA}_{\forall}~\mathrm{CWS}^{{\Xi}^q}$ is the difference of time-average advected heat UHA calculated following equation (1) in all CWS located in $q\textrm{th}$ quadrant Ξ per $i\textrm{th}$ LCZ during times *τ* when located upwind (*uw*) or downwind (*dw*) in comparison to the opposite quadrant.

\begin{equation*} {\Delta}\overline{T}_{\mathrm{CWS}_{\mathrm{LCZ}^{x}}^{i, {\Xi}^{q}}} = \overline{T_{\mathrm{CWS}_{\mathrm{LCZ}^{x}}^{i, {\Xi}^{q}}}^{{\tau}^{dw}} - T_{\mathrm{CWS}_{\mathrm{LCZ}^{x}}^{i, {\Xi}^{q}}}^{{\tau}^{uw}}} \end{equation*}

where ${\Delta}\overline{T}_{\mathrm{CWS}_{\mathrm{LCZ}^{x}}^{i, {\Xi}^{q}}}$ is the difference of time-average temperatures measured in all CWS located in $q\textrm{th}$ quadrant Ξ per $i\textrm{th}$ LCZ during times *τ* when located upwind (*uw*) or downwind (*dw*).

This way, we make sure to define the inter- and intra-LCZ differences in terms of heat advection. Since LCZ in London follow a relatively uniform concentric distribution, we are able to see where UHA is most important between the urban center and the suburbs.

## 3. Results

### 3.1. Quasi-climatology in the large domain (domain 1)

The detailed results of this analysis are given in supplementary information 5.2. In short, we find that there is a great level of spatial heterogeneity in the quasi-climatology of temperatures and daily temperature ranges that is not explained by the spatial heterogeneity of percentages of available measurements (Pearson’s *r* ^2^ $\lt$ 0.05). A consistent urban heat island of about ∼1.0 ^∘^C to ∼1.5 ^∘^C over the Greater London area appears. Measurements captured by CWS closer to the south coast are relatively hotter than inland ones during winter and cooler during summer. Daily temperature ranges are systematically lower in the denser parts of the Greater London area and on the south coast. This demonstrates the ability of CWS to capture spatial variability of local climates and supports the choice of Domain 2 for the subsequent UHA analysis (figure S1). We also find that CWS located in more compact and built-up LCZ are hotter on average and that they have lower daily temperature ranges throughout the year.

### 3.2. Influence of wind regime on urban heat heterogeneity and heat advection

**Figure 3.**
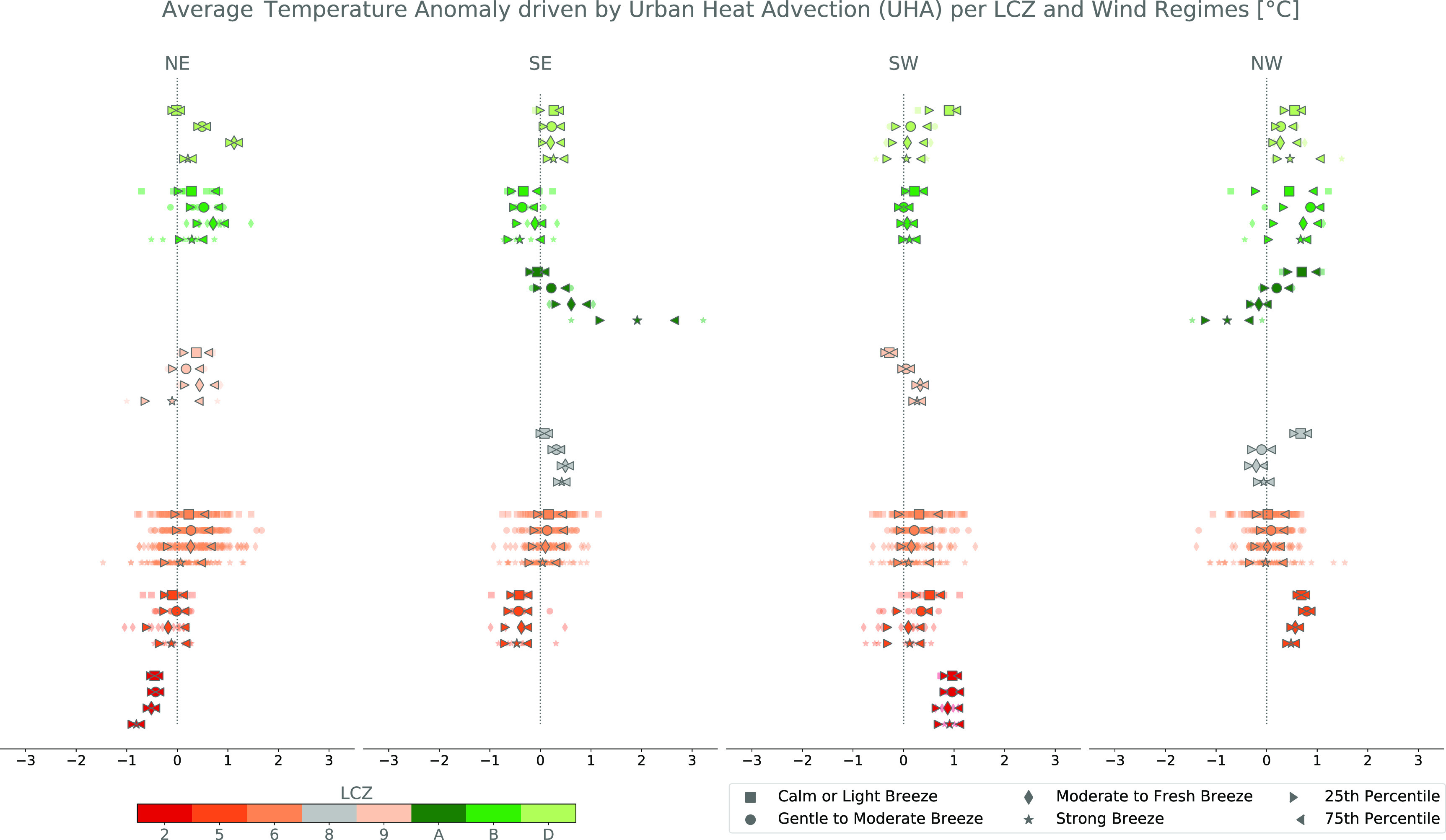
6-year average (2015–2020) hourly urban heat advection (UHA) per downwind citizen weather station (CWS) in each Local Climate Zone and upwind prevailing winds. Large markers represent the cross-CWS median of the average UHA and triangle whiskers represent the 25$\textrm{th}$ and 75$\textrm{th}$ percentiles. The vertical axis has no dimension. Daytime (7AM to 7PM) and nighttime (7PM to 7AM local time) plots are given in figures S9 and S10, respectively.

We find that winds have a noticeable impact on the temperature anomalies between similar LCZ located upwind or downwind. We calculate a cross-CWS time-mean positive UHA of 0.22 ± 0.96 ^∘^C over the 6-year period (2015–2020; figure 3). Maximum and minimum average UHA per CWS reach 1.57 ^∘^C and −1.21 ^∘^C, respectively. Depending on the wind strength, positive average UHA per CWS are usually between 0 ^∘^C and 1.0 ^∘^C, but can reach up to ∼3.0 ^∘^C, while negative average UHA per CWS rarely go below ${\sim}-1.0~^\circ$C, apart from the downwind stations in compact mid-rise (LCZ 2) and large low-rise (LCZ 8) during south- and north-easterly, and south-westerly conditions, respectively (figure 3). High degrees of intra-LCZ variability under different wind conditions are thus observed, meaning that while the median anomaly can be positive, certain CWS in an area with similar land-use land-covers will measure a negative anomaly. This could potentially be explained by important micro-scale effects, undetected by the simplified LCZ classification (Fenner *et al* 2017, Skarbit *et al* 2017, Varentsov *et al* 2021). Highest degrees of intra-LCZ variability are observed in open low-rise (LCZ 6), and more natural LCZ, like sparsely built (LCZ 9), dense trees (LCZ A), sparse trees (LCZ B) and low vegetation (LCZ D).

On average, CWS located in the same quadrant will experience a positive ΔUHA anomaly of 0.21 ± 0.13 ^∘^C when located downwind than upwind for each wind regime (table 1). When looking at intra-LCZ differences significant at 10% (table 1 in bold), CWS located in dense trees (LCZ A) from the south-eastern quadrant will observe a maximum ΔUHA of 0.49 ± 0.10 ^∘^C while the minimum of 0.07 ± 0.14 ^∘^C is observed for CWS located in sparse trees (LCZ B) of the same quadrant. A clear signal in heat advection among CWS located in natural LCZ is thus perceived. In urban LCZ, ΔUHA can reach 0.26 ^∘^C in open low-rise (LCZ 6). In addition, the inter-season and inter-LCZ variability of the anomaly appears to be more related to the direction of the prevailing winds and to their speeds rather than to the seasons (figure S8). This is also observable when looking at the temperature anomaly ΔT of the same CWS in each quadrant when located downwind or upwind (table 2). In fact, under northern wind conditions, CWS located in the Northern quadrants will be significantly cooler than during Southern prevailing winds (table 2 in bold). Noticeably, the opposite mostly happens in southern quadrants when subject to southern winds, but with no statistical significance. Only CWS of the south-western quadrant located in open low-rise (LCZ 6) and in sparse trees (LCZ B) were found to also be significantly cooler when located upwind.

**Table 1.** Average advected heat anomalies difference ΔUHA plus or minus standard deviation in degrees Celsius (^∘^C) per LCZ between same CWS in each quadrant when located upwind or downwind ($\Xi^{\mathrm{uw}}-\Xi^{\mathrm{dw}}$). Significance is given by the *p*-value from a one-sided dependent t-test with unequal variance. Statistically significant values at 10% are put in bold.

	North-East		South-East		South-West		North-West	
LCZ	ΔUHA	*p*-value	ΔUHA	*p*-value	ΔUHA	*p*-value	ΔUHA	*p*-value
Compact mid rise: LCZ 2	$-1.04 \times 10^{-3}\pm 0.26$	0.50	−0.23	—	−0.43	—	−0.03	—
Open mid rise: LCZ 5	$-0.07 \pm 0.31$	0.25	−0.23	—	$\boldsymbol{-0.10 \pm 0.10}$	$\boldsymbol{2.98 \times 10^{-3}}$	$-0.06 \pm 0.15$	0.19
Open low rise: LCZ 6	$\boldsymbol{-0.26 \pm 0.16}$	$\boldsymbol{1.22 \times 10^{-16}}$	$\boldsymbol{-0.16 \pm 0.16}$	$\boldsymbol{9.60 \times 10^{-13}}$	$\boldsymbol{-0.20 \pm 0.14}$	$\boldsymbol{1.84 \times 10^{-32}}$	$\boldsymbol{-0.12 \pm 0.14}$	$\boldsymbol{5.44 \times 10^{-10}}$
Large lowrise: LCZ 8	−0.41	—	$-0.22 \pm 0.19$	0.22	$-0.21 \pm 0.20$	0.13	−0.35	—
Sparsely built: LCZ 9	−0.44	—	$\boldsymbol{-0.20 \pm 0.10}$	$\boldsymbol{8.54 \times 10^{-3}}$	$-0.02 \pm 0.19$	0.45	—	—
Dense trees: LCZ A	—	—	$\boldsymbol{-0.49 \pm 0.10}$	**0.08**	$\boldsymbol{-0.12 \pm 0.11}$	**0.02**	$\boldsymbol{-0.26 \pm 0.07}$	**0.09**
Sparse trees: LCZ B	$\boldsymbol{-0.34 \pm 0.16}$	**0.01**	$\boldsymbol{-0.15 \pm 0.07}$	**0.04**	$\boldsymbol{-0.07 \pm 0.14}$	**0.07**	$\boldsymbol{-0.15 \pm 0.11}$	$\boldsymbol{6.78 \times 10^{-3}}$
Low plants: LCZ D	$\boldsymbol{-0.27 \pm 0.18}$	**0.04**	$-0.20 \pm 0.23$	0.17	−0.18	—	$\boldsymbol{-0.26 \pm 0.12}$	**0.04**

**Table 2.** Average temperature difference ΔT plus or minus standard deviation in degree Celsius (^∘^C) per Local Climate Zone between same citizen weather stations in each quadrants when located upwind or downwind ($\Xi^{\mathrm{uw}}-\Xi^{\mathrm{dw}}$). Significance is given by the *p*-value from a one-sided dependent t-test with unequal variance. Statistically significant values at 10% are put in bold.

	North-East		South-East		South-West		North-West	
LCZ	ΔT	*p*-value	ΔT	*p*-value	ΔT	*p*-value	ΔT	*p*-value
Compact mid rise: LCZ 2	$\boldsymbol{-2.0 \pm 0.56}$	$\boldsymbol{1.00 \times 10^{-3}}$	−0.37	—	0.52	—	−1.80	—
Open mid rise: LCZ 5	$\boldsymbol{-2.40 \pm 0.98}$	$\boldsymbol{8.01 \times 10^{-6}}$	−0.37	—	$0.51 \pm 0.81$	0.97	$\boldsymbol{-1.88 \pm 0.30}$	$\boldsymbol{2.41 \times 10^{-6}}$
Open low rise: LCZ 6	$\boldsymbol{-1.87 \pm 0.66}$	$\boldsymbol{6.68 \times 10^{-28}}$	$\boldsymbol{-0.16 \pm 0.59}$	**0.01**	$0.53 \pm 0.71$	0.99	$\boldsymbol{-2.10 \pm 0.34}$	$\boldsymbol{8.69 \times 10^{-59}}$
Large lowrise: LCZ 8	−4.11	—	$-0.07 \pm 0.36$	0.44	$0.58 \pm 0.31$	0.94	−2.05	—
Sparsely built: LCZ 9	−2.13	—	$-0.13 \pm 0.49$	0.31	$0.17 \pm 0.73$	0.57	—	—
Dense trees: LCZ A	—	—	$-0.09 \pm 0.09$	0.25	$3.60 \times 10^{-3}\pm0.60$	0.50	$\boldsymbol{-2.34 \pm 0.24}$	**0.03**
Sparse trees: LCZ B	$\boldsymbol{-2.47 \pm 0.48}$	**0.06**	$\boldsymbol{-0.56 \pm 0.40}$	**0.09**	$0.76 \pm 0.37$	0.99	$\boldsymbol{-2.39 \pm 0.24}$	$\boldsymbol{1.48 \times 10^{-7}}$
Low plants: LCZ D	$\boldsymbol{-2.01 \pm 1.25}$	**0.03**	$-0.12 \pm 0.58$	0.40	0.60	—	$\boldsymbol{-2.52 \pm 0.56}$	**0.01**

**Figure 4.**
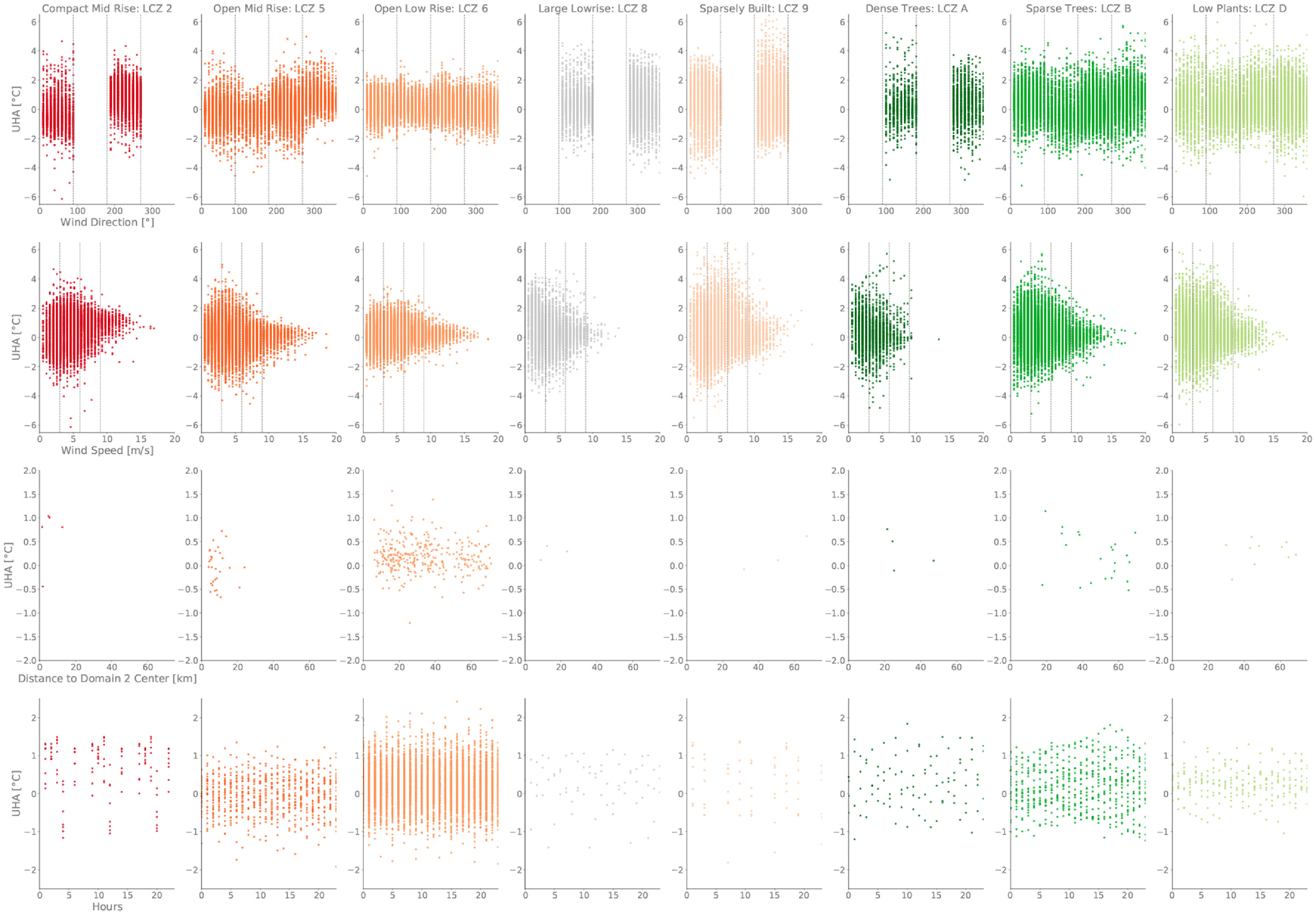
Hourly cross-CWS average UHA against wind speed and direction (first two rows above; dotted lines represent the limits for their respective classes), time-mean UHA per CWS against distance to the center of domain 2 (third row) and 6-year-hourly-mean UHA per CWS (fourth row). Each row is subdivided by LCZ to allow for inter- and intra-LCZ comparison.

**Figure 5.**
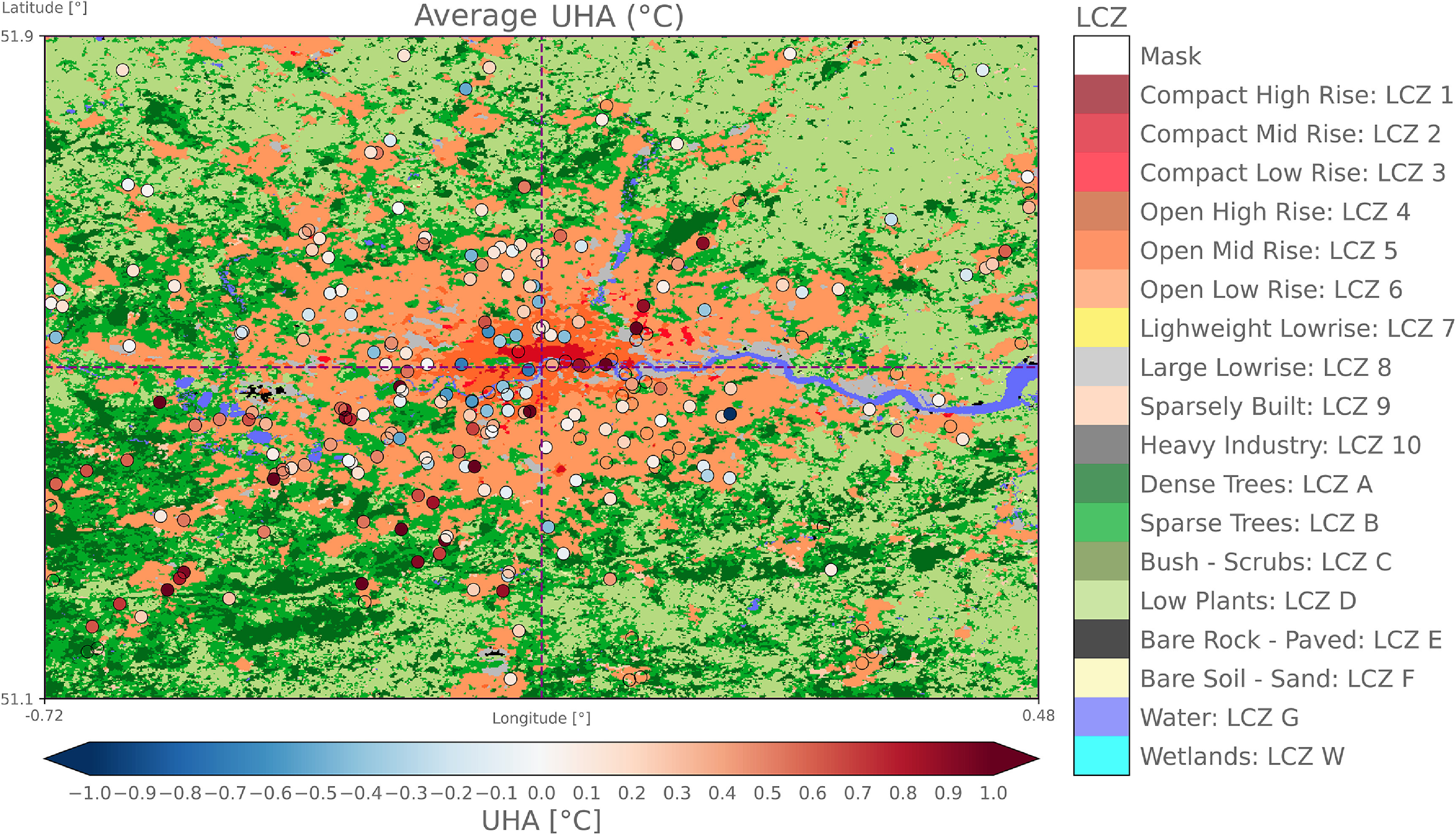
6-year average urban heat advection per citizen weather stations overlaid on the Local Climate Zones map of the Greater London area (domain 2). Dashed purple lines represent the quadrants borders. CWS where no UHA could be measured are made transparent.

Stronger winds are not related to higher heat transport. On the contrary, they tend to reduce the amount of advected heat in certain LCZ, like open mid-rise (LCZ 5) during south-westerly conditions (figures 3 and S8). In addition, the higher the wind speed, the lower the inter-CWS variability of UHA and the more it converges towards the average UHA, suggesting micro-scale effects are of lesser importance (figure 4, second row). Openly built urban and natural LCZ appear to be similarly affected by UHA (figure 3). CWS located in compact mid-rise (LCZ 2) or large-lowrise (LCZ 8), mostly located in the center of Domain 2, show large fluctuations of UHA from one wind condition to another (figure 3). In contrast, UHA in more open LCZ, like open mid- or low-rises (LCZ 5 and LCZ 6, respectively) are similar for all wind regimes. Importantly, negative UHA values are found, suggesting that UHA does not happen homogeneously across the downwind quadrants. By looking at the temporal evolution of UHA, we did not find a systematic signal showing that UHA is more pronounced whether during daytime (7AM to 7PM)/nighttime (7PM to 7AM) or more specific hours (figure 4, fourth row; figures S9 and S10). Trying to relate UHA to the distance to the city center and to the wind direction was inconclusive because of the great inter-CWS variability in UHA. Indeed, CWS close to the urban center, mostly composed of compact and open mid-rises (LCZ 2 and LCZ 5), and located in the south-western corner show a negative 6-year averaged UHA (figure 5). UHA appears to be more pronounced in peripheric areas and along the south-west/north-east transect. Mostly positive UHA is found in each quadrant although the inter-CWS variability varies greatly from one quadrant to another. For instance, lowest variability in average UHA is found in the north-western quadrant, while higher levels of inter-CWS variability are found in the south-western and north-eastern quadrants.

## 4. Discussion and conclusions

In this paper, we show that crowdsourced CWS can help with monitoring and studying urban temperatures for recent years in a variety of urban environments, supporting a number of previous studies (e.g. Meier *et al* 2015, 2017, Chapman *et al* 2017, Fenner *et al* 2017, 2019, Napoly *et al* 2018, Droste *et al* 2020, Varentsov *et al* 2020, Venter *et al* 2020, 2021, de Vos *et al* 2020, Benjamin *et al* 2021, Potgieter *et al* 2021). In our study, quality-checked CWS are sensitive to seasonal changes and local climate features in the south-eastern parts of England, like the coastal climate in the South, or the heterogeneity of the urban environments in the Greater London area—in terms of Local Climate Zones (LCZ).

By focusing on a 6-year period ranging from 2015 to 2020, we showed a marked higher urban heat in the Greater London area by 1.0 ^∘^C–1.5 ^∘^C using CWS. This was also described by Chandler (1965) for the 1921–1950 period with an average difference of 1.1 ^∘^C and 0.55 ^∘^C between *surrounding country* and *suburbs*, respectively, and the *central districts*. This confirms that London’s urban heat island intensity has not changed markedly over recent decades (Bassett *et al* 2021), even if it has been subject to recent changes in temperatures related to climate change (see IPCC report; Pachauri *et al* 2014). Urban heat is however related to land-use/land-covers, with more central compact mid rises (LCZ 2) always revealing an increased monthly average hourly temperature by up to ∼1.5 ^∘^C throughout the year compared to more open LCZ, and a smaller daily temperature range. The urban heat magnitude monitored by the CWS is in line with recent observational and modelling studies, although most of them focused on summer months only (Mavrogianni *et al* 2011, Grawe *et al* 2013, Chapman *et al* 2017, Benjamin *et al* 2021). Intra-urban heterogeneity using LCZ was also demonstrated in previous studies from Fenner *et al* (2017), Benjamin *et al* (2021), Potgieter *et al* (2021)and Varentsov *et al* (2021). Similar differences between more compact LCZ and more open or natural ones were found in Berlin for the year 2015 only (Fenner *et al* 2017). Nonetheless, since cities usually have a denser urban center, it is difficult to attribute which part of the positive anomaly is related to the urban typology rather than to the central location subject to heat advection from the surrounding environments. Varentsov *et al* (2021) found in Moscow, Russia, that meso-scale urban surroundings are about as equally important as local-scale surroundings for explaining the spatial heterogeneity of urban heat. Potgieter *et al* (2021) emphasized the latter point by discussing why more densely built high-rises in the city of Sydney, Australia, may show cooler minima than other urban environments in a coastal climate.

**Figure 6.**
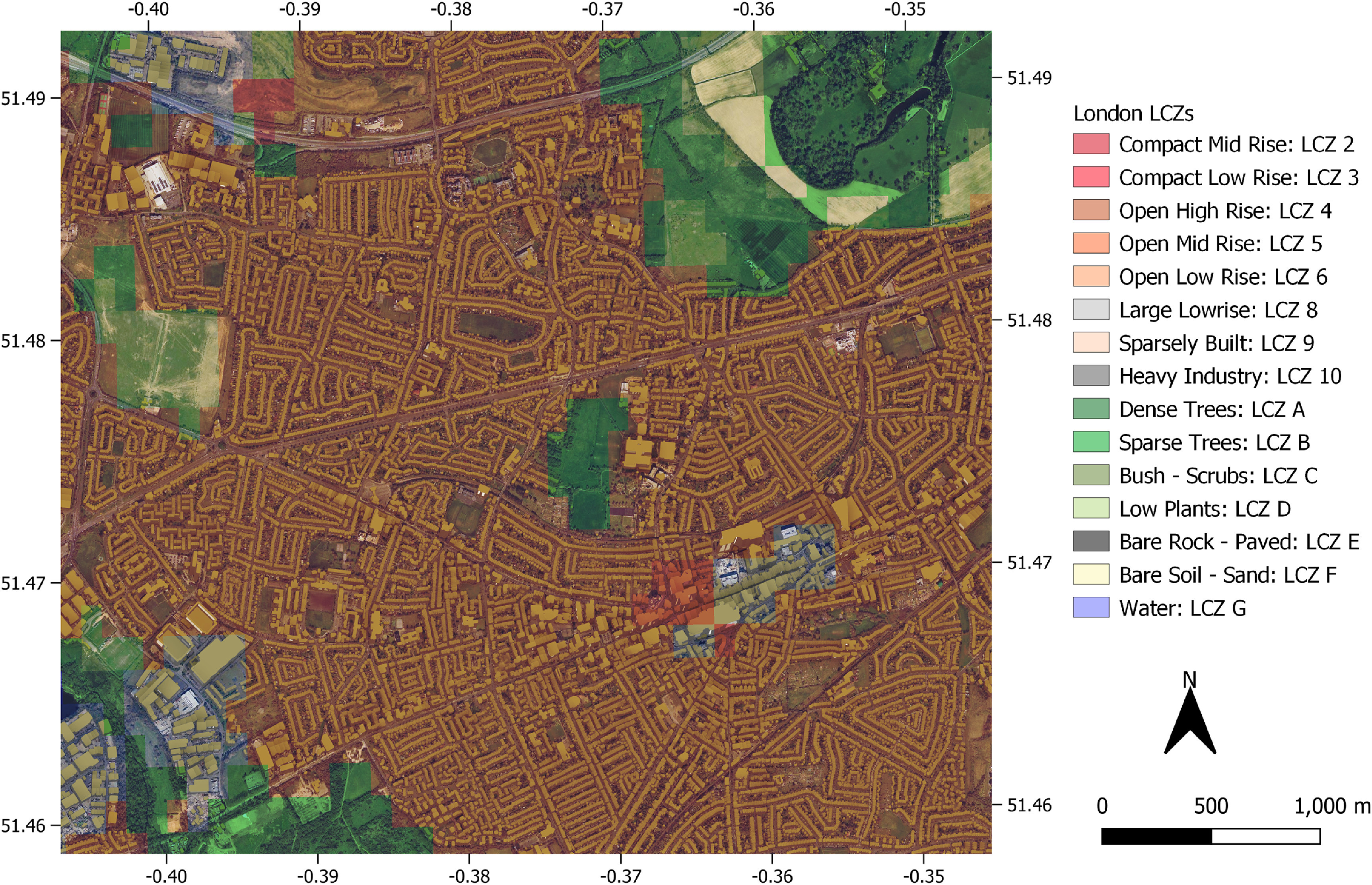
Example of the built-up environment’s three-dimensional complexity in the city of London overlaid on the European LCZ map by Demuzere *et al* (2019). Coordinates are in longitude and latitude.

In our study, we showed that prevailing winds defined at the Heathrow station can explain differences in temperatures at the same CWS location and drive urban heat advection (UHA) in Greater London. The latter is significantly captured by CWS. On average, UHA transfers London’s central neighborhoods’ urban heat to more suburban areas. This advection is around 0.22 ± 0.96 ^∘^C on average in all Greater London neighbourhoods for all downwind wind conditions during our 6-year period (2015–2020). Natural areas within London are also subject to urban heat transport, which could reduce their value as urban cool spots. In general, UHA heats downwind quadrants and brings cooler air from the rural lands on upwind quadrants. This causes an important spatio-temporal variability of urban heat. Besides, we found high degrees of intra-LCZ variability in UHA—e.g. in open mid- or low-rise (LCZ 5 and LCZ 6) locations. In fact, depending on the wind conditions and the LCZ in which CWS are located, UHA can reach up to ∼3 ^∘^C on average with values usually below 1.0 ^∘^C—being in line with previous studies on UHA in Birmingham by Bassett *et al* (2016) and Heaviside *et al* (2015). At certain hours in our 6-year period, we could even measure positive and negative UHA of up to ∼6.0 ^∘^C. This variability in hourly UHA tended to be reduced with higher wind speeds (e.g. above 9 m·s^−1^). It can be explained by the heterogeneous and complex diffusion of airflows within the urban three-dimensional environment, as illustrated in figure 6 (Hall *et al* 1997, Grimmond and Oke 1999, Oke *et al* 2017). Such results actually suggest that although general meteorological circulations can generally explain where heat will be transported, more local micro-climatic phenomena are of equal importance in explaining the spatio-temporal variability of UHA. The latter also explains why both negative and positive UHA can be measured at the same time by CWS in downwind quadrants. More in-depth studies using machine learning and trying to relate other surface earth observations to air-temperature variations should be attempted to explain the variability of urban heat (e.g. Venter *et al* 2021) and the physical mechanisms behind UHA.

Despite the novelty of the results we show, our study suffers from certain limitations. For instance, we: (a) used partly arbitrary wind regime classes derived from the Beaufort scale and quantiles of wind speeds that could be refined via more quantitative analysis; (b) considered quadrants with very few CWS to still be representative of the heat advection because of the restrictions we imposed in the data selection; (c) did not take into consideration the location of the CWS within their LCZ at 100 m horizontal resolution; (d) did not estimate how surrounding LCZ may influence the measurement taken at a certain CWS location; (e) used the previous version of the CrowdQC quality check by Grassmann *et al* (2018) that has since been improved (Fenner *et al* 2021); (f) did not study the vertical winds and the related heat advection; and (g) only used one representative official weather station (Heathrow Airport) to classify prevailing winds over the area of interest. The latter can be considered as the main limitation of this study. We found nonetheless that hourly prevailing winds characterized at Heathrow were originating from the same quadrant 75% of the time when compared to Kenley Airfield and Kew Gardens, and 78% of the time for Northolt. Western stations (Heathrow, Northolt and Kew Gardens) were agreeing 67% of time, and all stations 57% of it. This illustrates the complex dynamics and physics that can play a role in the dispersion of the urban heat plume (Oke 1982, Souch and Grimmond 2006, Heaviside *et al* 2015, Oke *et al* 2017). Hence, future observational and modelling studies are required to better understand the spatio-temporal patterns of UHA.

In particular, we recommend future studies to improve our understanding of the physical mechanisms driving UHA and to study the relevance of using CWS to do this. Future research should: (a) estimate the representativity of measured temperatures in each LCZ depending on the location of CWS over rasterized LCZ land use/land cover maps; (b) evaluate, for example through machine learning, the impact of surrounding environments at varying distances (e.g. LCZ, water, forests$\ldots$) on the urban heat and its advection; (c) quantify the spatio-temporal distribution of UHA depending on specific synoptic conditions when urban heat is more or less pronounced; (d) deepen the investigation on the diurnal evolution of UHA to see if UHA follows a predictable diurnal pattern correlated to the urban heat island intensity, for example; (e) improve the analysis on the seasonality of wind regimes and UHA to see if the necessary strength to advect heat is related to the magnitude of the urban heat in different seasons; and (f) take benefit from the recently densifying network of CWS anemometers (Droste *et al* 2020, Chen *et al* 2021) and the other existing AWS to more specifically study the spatial patterns of UHA in relation to the micro-climatic dynamics and local prevailing winds.

## Data Availability

The data generated and/or analysed during the current study are not publicly available for legal/ethical reasons but are available from the corresponding author on reasonable request.

## Data and scripts accessibility

The data cannot be shared publicly for policy reasons. It can be sent upon reasonable request to the corresponding author. MIDAS data was retrieved at https://doi.org/10.5285/3bd7221d4844435dad2fa030f26ab5fd. The SRTM digital elevation model was retrieved on the Google’s Earth Engine (more info: https://developers.google.com/earth-engine/datasets/catalog/USGS_SRTMGL1_003?hl=en#description). The European Local Climate Zones map can be obtained at the following link thanks to the WUDAPT project: https://figshare.com/articles/dataset/European_LCZ_map/13322450. All scripts used for the data acquisition (Netatmo), treatment and analysis are available at this GitHub repository: https://github.com/oscarbrousse/UHA_CWS_ERL2022. A README file explaining the workflow to be followed for reproducing the results is given in the repository.
